# High-Sensitivity Troponin T as a Prognostic Factor of Conventional Echocardiographic Parameters in Cancer Patients: A Prospective Observational Study

**DOI:** 10.3390/medicina61111911

**Published:** 2025-10-24

**Authors:** Svetoslava Elefterova Slavcheva, Sevim Ahmed Shefket, Yana Bocheva, Atanas Angelov

**Affiliations:** 1 First Department of Internal Diseases, Faculty of Medicine, Medical University “Prof. Dr. Paraskev Stoyanov”, 9000 Varna, Bulgaria; atanas.a.atanasov@mu-varna.bg; 2 First Cardiology Clinic with Intensive Care Unit, St. Marina University Hospital, 9000 Varna, Bulgaria; 3 Department of Clinical Laboratory, Faculty of Medicine, Medical University “Prof. Dr. Paraskev Stoyanov”, 9000 Varna, Bulgaria; sevim.shefket@mu-varna.bg (S.A.S.); yana.bocheva@mu-varna.bg (Y.B.); 4 Clinical Laboratory, St. Marina University Hospital, 9000 Varna, Bulgaria

**Keywords:** cardiotoxicity, echocardiography, biomarkers, high-sensitivity troponins, anthracycline, trastuzumab

## Abstract

*Background and Objectives*: Cardiac injury caused by cancer therapy can be detected early using high-sensitivity cardiac troponins (hs-cTns), and this is crucial for preventing irreversible consequences. Clinically relevant issues regarding hs-cTns in oncologic settings—such as reliable cut-off values, the optimal assessment timeframe, factors influencing their levels, and their prognostic ability in relation to functional echocardiographic parameters—require further investigation. In this study, we aimed to examine the determinants of hs-cTnT variations during cancer therapy and the relationship between the biomarker and functional conventional echocardiographic parameters. *Materials and Methods*: We prospectively evaluated adult patients scheduled for chemotherapy for either breast or gastrointestinal cancers, excluding those with pulmonary and cardiac disorders. We enrolled 40 patients who underwent a minimum of one cycle of potentially cardiotoxic regimens containing at least one of the following agents: anthracyclines, cyclophosphamide, taxanes, 5-fluorouracil, platinum compounds, trastuzumab, or bevacizumab. We observed two-dimensional and tissue Doppler echocardiographic parameters and hs-cTnT levels for a median of 360 days (IQR 162, 478) following the start of chemotherapy. *Results*: The generalised estimating equation (GEE) analysis revealed significant elevations in hs-cTnT levels at three months (β = 1.2; *p* = 0.005) and six months (β = 2.3; *p* = 0.02) from baseline, influenced by anthracycline treatment (*p* = 0.009), renal function (*p* = 0.003), and increased cardiotoxicity risk (high: *p* = 0.013; medium: *p* < 0.001). Elevated hs-cTnT levels independently predicted the deterioration of the LV longitudinal myocardial function, measured by the systolic tissue velocities, according to the GEE analysis. The receiver operating characteristic curve-derived hs-cTnT thresholds—of 8.23 ng/L and 8.08 ng/L—had a high negative predictive value for identifying Average and Lateral LVS′ decreases, respectively. *Conclusions*: Our research supports the use of baseline and continuing hs-cTnT testing in cancer patients, showing the dependence of the biomarker on renal function, cardiovascular toxicity risk level, and anthracycline treatment. The hs-cTnT cut-off value of approximately 8 ng/L may suggest a low probability of longitudinal myocardial function impairment and this observation needs further validation in larger cohorts.

## 1. Introduction

Cancer therapeutics may affect myocardial function through a variety of mecha-nisms [1]. Anthracyclines, trastuzumab, 5-fluorouracil, cyclophosphamide, and vascular-endothelial growth factor (VEGF) inhibitors can cause direct cardiomyocyte damage. Vascular toxicity, myocardial ischaemia and inflammation are mechanisms that indirectly injure cardiac cells and are induced by platinum compounds, taxanes, cyclophosphamide, 5-fluorouracil (5-FU), VEGF inhibitors, and immunotherapeutics [1]. Irrespective of the processes behind cardiomyocyte damage, it may lead to the release of cardiac troponin into the bloodstream.

Timely identification of cardiotoxicity remains crucial for averting irreversible cardiac injury [2]. Cardiac troponins are well-established biomarkers for detecting cancer therapy-related cardiac injury before it becomes visible through imaging modalities [3]. The biomarkers have been studied in relation to various antineoplastic regimens, including anthracyclines, targeted therapy, and immune checkpoint inhibitors [3]. In cancer patients, cardiac troponins—assessed through conventional assays—can predict the development of cardiac dysfunction and other cardiovascular events, such as cardiac death, pulmonary oedema, heart failure, rhythm disorders, and failure to recover after the onset of cardiotoxicity [4,5,6]. According to the current cardio-oncology guidelines, the biomarkers serve as a determinant of both baseline and ongoing cardiotoxicity risk related to cancer therapy [7].

Using high-sensitivity cardiac troponin (hs-cTn) assays, earlier identification of cardiotoxicity appears to be even more reliable. The sensitivity of the assays poses an issue; they are capable of detecting minimum troponin concentrations that may be influenced by the cancer itself and additional factors—such as inflammation and renal impairment—that are prevalent among cancer patients [8,9]. Although increasing evidence suggests that hs-cTns in cancer populations can predict all-cause mortality and left ventricular ejection fraction (LVEF) decline, little is known about the prediction of other systolic and diastolic functional echocardiographic parameters [10,11,12,13,14,15]. Ongoing research continues to focus on determining the optimal sample timing and the most effective hs-cTn cut-offs for accurately predicting cardiotoxicity onset [7,10,11,13,14,16,17,18,19,20,21,22]. The varying sensitivity of different assay types in detecting myocardial necrosis further complicates this endeavour [23].

In this study, we aimed to evaluate variations in hs-cTnT levels during potentially cardiotoxic systemic cancer therapy and identify factors influencing biomarker changes in an oncological context. Additionally, we sought to investigate the prognostic relationship between hs-cTnT and conventional echocardiographic functional parameters of the left and right ventricles.

## 2. Materials and Methods

### 2.1. General Proceedings

We performed a prospective observational study on patients aged over 18 who provided informed consent and scheduled to receive chemotherapy for either breast or gastrointestinal malignancies. The research was conducted at the First Cardiology Clinic at St. Marina University Hospital, Varna, Bulgaria, from June 2019 to February 2024. The patients were recruited consecutively among those referred for cardiologic evaluation from the Clinic of Medical Oncology at the same hospital, and if deemed eligible, were enrolled in the study. Patients were eligible if they underwent at least one cycle of potentially cardiotoxic chemotherapy that included one of the following agents: anthracyclines, 5-fluorouracil, platinum compounds, or taxanes, whether or not accompanied by tar-geted therapy with trastuzumab or bevacizumab. Patients who had undergone chem-otherapy or radiotherapy more than five years ago and did not receive continuous hormonal or targeted therapy were also included in the study. The exclusion criteria were as follows: a history of persistent pulmonary conditions and pulmonary embolism; haemodynamically relevant valvular disorders; persistent atrial fibrillation; heart failure, left ventricular systolic dysfunction, and established coronary artery disease; a poor acoustic window; refusal to participate in the study and attendance to less than three follow-up visits.

Our team performed baseline clinical, echocardiographic, and laboratory assessments before chemotherapy and at follow-up visits up to 18 months (at 1, 3, 6, 9, 12, and 18 months) after oncological treatment initiation. We compared the values of key echocardiographic and laboratory measures (creatinine, haemoglobin and hs-cTnT) between each time point and the baseline. The Research Ethics Committee of the Medical University of Varna approved the study protocol (approval number 84/27 June 2019). Cardiotoxicity risk was estimated for patients receiving anthracyclines, human epidermal growth factor receptor 2 (HER2)-targeted therapy, VEGF inhibitors. We used cardiovascular risk stratification tools from the Heart Failure Association and International Cardio-Oncology Society (HFA-ICOS) recommendations [7,24]. Cardioprotective medications renin–angiotensin–aldosterone system (RAAS) inhibitors or beta blockers) might have been initiated (without blinding) at some point of observation at the discretion of the cardiologist.

### 2.2. Echocardiographic Evaluation

Echocardiographic examinations were carried out by a single certified cardiologist using a GE Vivid 7 Pro ultrasound system. We monitored conventional two-dimensional and tissue Doppler echocardiographic parameters. Speckle-tracking echocardiography (STE) equipment was unavailable at our research centre, which impeded the assessment of deformational indices, particularly the global longitudinal strain (GLS). The evaluation of echocardiographic parameters adhered to the guidelines of the American Society of Echocardiography and the European Society of Cardiovascular Imaging [24]. Final values for all the echocardiographic parameters represented the average of at least three consecutive measurements to mitigate intraobserver variability.

Left Ventricular (LV) Assessment: We evaluated indexed end-diastolic volume (LVEDVi), LVEF by biplane Simpson’s method, mitral annular plane systolic excursion averaged of the obtained values at the lateral and septal annulus (MAPSE), and Tei index with PW Doppler (LVMPI-PW). Left ventricular filling parameters included mitral early (E) and late (A) inflow velocities, E/A ratio, isovolumic relaxation time (IVRT), and deceleration time (DecT). To assess longitudinal myocardial function, we tracked peak systolic tissue velocities of the left ventricle, following imaging guidelines when GLS measurement is not feasible [24]. Peak tissue Doppler systolic S′ velocities were obtained at the septal mitral annulus (Septal LVS′), lateral mitral annulus (Lateral LVS′) and the average of the two (Average LVS′). Early diastolic tissue Doppler e’ velocities (average of the septal and lateral e′) (LVe′), E/e′ ratio, valve function, and left atrial volume (LAV) were calculated.

Right Ventricular (RV) Assessment: We measured indexed RV end-diastolic area (RVA) for body surface area; right ventricular fractional area changes (RVFAC); tricuspid annular plane systolic excursion (TAPSE); systolic S′ (RVS′) tissue Doppler velocity at the lateral tricuspid annulus; Tei index with tissue Doppler (RVMPI-TDI); tricuspid regurgitation; and right atrial volume (RAV).

### 2.3. Definitions and Thresholds for Echocardiographic Parameters

Similar to the definition of asymptomatic cancer therapy-related cardiac dysfunction (CTRCD) in the 2022 European Society of Cardiology (ESC) guidelines on cardio-oncology, we used Average LVS′ as a surrogate of LV global longitudinal strain to evaluate longitudinal LV function [7,24]. Pathological decline in LV systolic tissue velocities was defined as a relative drop exceeding 15% from the baseline, with the following thresholds: Septal LVS′ ≤ 6 cm/s; Lateral LVS′ ≤ 8 cm/s; Average LVS′ ≤ 7 cm/s. The minimal cut-off values for systolic tissue LV velocities were established to be below the normative mean values across all age groups in the population [25].

We defined LV systolic dysfunction due to oncological treatment as either a relative reduction in LVEF exceeding 10% to levels below 50% and/or a relative decrease in Average LVS′ greater than 15% to values ≤ 7 cm/s. The pathological threshold for RVFAC was determined as a relative decline exceeding 20%, resulting in a value of ≤35%. For RVS′, a relative reduction greater than 15% to a value of ≤9.5 cm/s was considered pathological.

### 2.4. High-Sensitivity Cardiac Troponin T Measurements

The biochemical marker, high-sensitivity cardiac troponin T (hs-cTnT), was analysed in our institutional laboratory. The samples were collected at baseline and before chemotherapy cycles during the first- and third-month visits, with additional sampling performed when clinically indicated. We implemented the electrochemiluminescence immunoassay method using the Cobas analysers of the Roche company. The maximum threshold of the hs-cTnT was 14 ng/L, and the lower detection limit was 3 ng/L.

### 2.5. Statistical Methods

Statistical analyses were performed using R software, version 4.3.2 (31 October 2023). Descriptive statistics were employed to calculate the mean, standard deviation (SD), median, interquartile range (IQR), and proportions, depending on the type and distribution of the variables. To compare correlated variables between two time intervals, paired *t*-tests or Wilcoxon signed-rank tests were applied contingent on the data distribution.

We used a generalised linear model (GLM) to graphically present the time trend of hsTnT changes. For an accurate assessment of the temporal change in hsTnT, we em-ployed a repeated-measures regression analysis using a generalised estimating equation (GEE). Repeated-measures GEE analysis is appropriate for observational studies and can estimate correlated continuous (with normal or skewed distribution) and bi-nary response variables.

We conducted a GEE linear regression univariate analysis to identify the factors that contribute to the variability of hsTnT. After reviewing the literature, we decided on the following confounders for analysis: age, cancer therapy (anthracycline-based, trastuzumab), cardioprotective treatment, haemoglobin, cancer stage, estimated glomerular filtration rate (eGFR), and HFA-ICOS cardiotoxicity risk. The significant and borderline significant factors were included in a GEE multivariate linear regression model, from which age was omitted, as it was incorporated in eGFR and HFA-ICOS cardiotoxicity risk.

We also conducted univariate and multivariate logistic regression GEE analyses to determine the prognostic value of hs-cTnT in relation to alterations in echocardiographic parameters. In addition to hs-cTnT, we included the following potential factors into the multivariate analysis: age, cancer stage, type of systemic cancer therapy, cardiotoxicity risk level, and cardioprotective treatment.

Receiver operating characteristic (ROC) analysis was employed to assess the diagnostic performance of hs-cTnT in detecting pathological deviations in specific echocardiographic variables. For the ROC analysis, we used all the values of hs-cTnT, sampled at both the baseline and follow-up visits. Statistical significance was defined as *p* < 0.05.

We performed a power analysis using the rate (42.8%) of elevated hs-cTnT levels in the largest study (323 breast cancer patients) that estimated the biomarker in a population more similar to our research in terms of antineoplastic medications—anthracycline, trastuzumab, cyclophosphamide, taxanes, and platinum—as well as a similar sample collection timing [19]. As our investigation excluded patients with known cardiac disease and included patients with gastrointestinal cancers, we anticipated a lower rate (20%) of abnormally high hs-cTnT levels. The estimated power was 0.8 for a sample size of 39 and an alpha level of 0.05 for type I and II errors.

## 3. Results

### 3.1. Demographic and Clinical Characteristics of the Study Population

The final cohort comprised 40 individuals, predominantly women (Table 1). The patients’ age ranged from 31 to 74 years. Table 1 presents the demographic and clinical characteristics of the study population. Only a minor proportion of the participants had diabetes or dyslipidaemia, while nearly half of them had hypertension. No patient had a very high HFA-ICOS risk level, and half of the population had a low HFA-ICOS risk. Approximately one-third of the patients were receiving cardioprotective medication before enrolling in the study. Twenty per cent of the population commenced cardioprotection during the observation period. The median duration of patient monitoring was approximately one year, with a median of four follow-up visits.

### 3.2. Cancer-Related Characteristics

Breast cancer was present in three-quarters of the patients, while gastrointestinal malignancies were diagnosed in the remaining patients. Table 2 summarises the cancer-related disease characteristics and therapeutic interventions. All patients received combined chemotherapy. Approximately 50% underwent 4 to 6 cycles of anthracycline-based treatment at a relatively low cumulative dose—the estimated mean doxorubicine equivalent dose, indexed for BSA was 123 (SD 43) mg/m. A quarter of the patients received HER2-targeted therapy, primarily following non-anthracycline-based chemotherapy. One-third of the population received treatment with non-anthracycline regimens, which included at least one of the following: taxane, platinum, and 5-fluorouracil. Figure S1 illustrates the distribution of various systemic therapies by cancer type, taking into account the high risk of cardiomyopathy associated with anthracyclines and HER2-targeted treatments. Table S1 presents the chemotherapy regimens used for cancer treatment in the population. One-fifth of the patients had left-chest radiation therapy, administered post-chemotherapy.

### 3.3. Echocardiographic Evaluation and Clinical Events

Table 3 and Table S2 present baseline dimensional and functional echocardiographic parameters of the left and right ventricles. No patient had left ventricular dysfunction at baseline assessment. During the observation, all right and left ventricular functional indices showed statistically significant deviations, as shown in Table 3. The median time for maximum deviations in all functional echocardiographic parameters was within 3 to 6 months post-treatment initiation. Eight (20%) patients developed LV systolic dysfunction, identified through decreased LVEF in one patient at the 6th-month visit, decreased Average LVS′ in five patients between the 1st and 9th months, and a combination of both parameters in two patients at the 3rd and 6th months. One patient exhibited symptoms consistent with heart failure.

During the follow-up, one patient exhibited symptoms consistent with NYHA functional class II heart failure, which did not require hospitalisation; this patient also presented with LV systolic dysfunction. Two patients died: one from cancer progression and one from pneumonia. After five cycles of chemotherapy with 5-FU and oxaliplatin for colon cancer, one patient developed right leg deep vein thrombosis, necessitating the cessation of the cancer treatment and ambulatory anticoagulation. One patient developed hypothyroidism following seven cycles of a breast cancer regimen including the immune-checkpoint inhibitor pembrolizumab, epirubicin, cyclophosphamide, carboplatin, and paclitaxel.

### 3.4. High-Sensitivity Cardiac Troponin Evaluation

We monitored hs-cTnT concentration in the majority of patients up to the third month to facilitate early detection of myocardial damage. Table 4 presents the median biomarker values at each follow-up stage and the corresponding number of examined patients.

**Figure 1:**
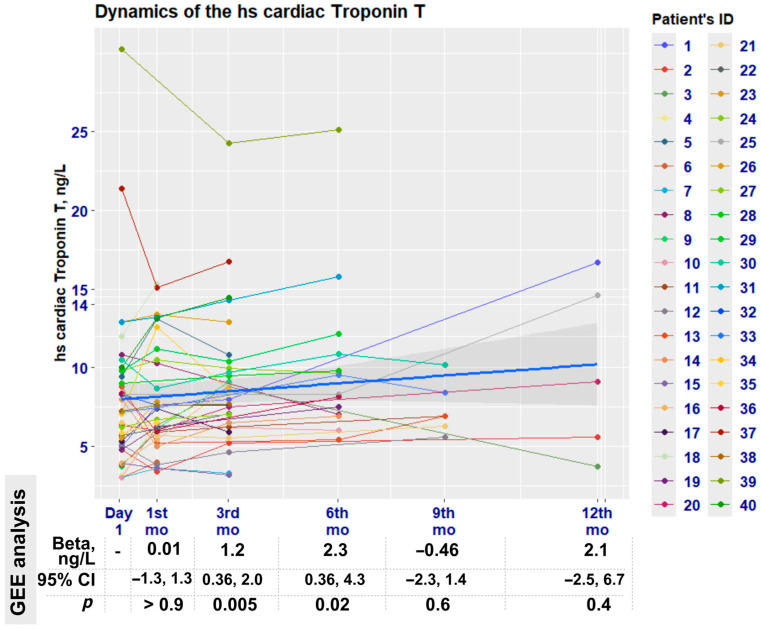
Graphical representation of the temporal changes in the hsTnT levels. The blue line represents overall biomarker variation with 95% confidence intervals (GLM analysis) shown in grey. Different colours indicate parameter value variances for each patient. Quantitative assessment of parameter changes for each visit, based on GEE analysis, is presented below the graphics. The figure was created using R and Biorender.com.

The GEE regression analysis of the biomarker dynamics (Figure 1) showed a statistically significant increase in its levels at the third (*p* = 0.005) and sixth (*p* = 0.02) months.

A total of five patients (12.5%) reached pathological maximal levels of hs-cTnT (exceeding 14 ng/L), with baseline values within the normal range (Figure S2). The mean difference between the baseline and peak hs-cTnT was 1.85 ng/L (SD 3.0). The median relative percentage increase in hs-cTnT was 23.2% (IQR 3.8%, 47%). None of the five individuals with biomarker concentration exceeding the upper limit of normal demonstrated left ventricular or right ventricular systolic dysfunction, although they approached the pathological thresholds of the LV and RV functional parameters (Figure S3). One patient exhibited symptoms compatible with heart failure. Three patients were treated with anthracycline-based regimens, one with trastuzumab and one with sequential anthracycline chemotherapy and trastuzumab. Elevation of hs-cTnT occurred in two patients at the 1-year visit in the trastuzumab therapy setting. In the other three patients, treated with anthracyclines, the abnormal hs-cTnT was detected at the 1-month and 3-month visits. Two patients (5%) had elevated baseline hs-cTnT > 14 ng/L (Figure S2), both with advanced cancer stage (IIIB and IIIC).

### 3.5. Determinants of hs-cTnT Variability During Oncological Treatment

**Figure 2:**
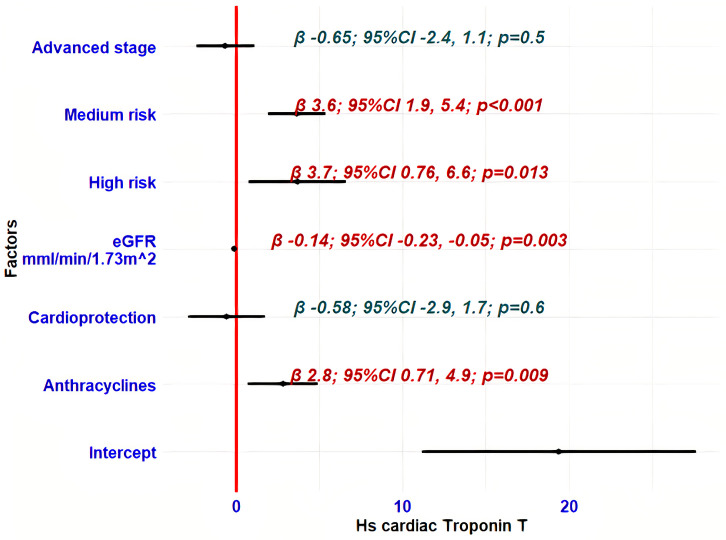
Forest plot of multivariate generalised estimating equations linear regression analysis for hs-cTnT change prediction.

We selected several factors—age, haemoglobin, estimated glomerular filtration rate (eGFR), type of cancer therapy, cardioprotective drugs, cancer stage, and HFA-ICOS cardiotoxicity risk—to examine their impact on the hs-cTnT deviation as a longitudinal variable. Univariate GEE linear regression analysis indicated that hsTnT levels increased with advancing age, higher cardiotoxicity risk, advanced cancer stage, and a decreasing eGFR (Table 5). These factors were selected for the multivariate analysis (Figure 2), except for age, which is incorporated in the HFA-ICOS risk level and glomerular filtration rate calculation. Cardioprotective and anthracycline therapies demonstrated borderline statistically significant influence on hs-cTnT release (Table 5) and were also incorporated in the multivariate regression model (Figure 2).

Multivariate GEE regression analysis identified cardiotoxicity risk level, anthracycline-based treatment, and glomerular filtration rate as independent predictors of hs-cTnT increase (Figure 2). Cancer stage had no independent statistical predictive value.

### 3.6. High-Sensitivity Cardiac Troponin T as a Prognostic Indicator of Left and Right Ventricular Function

We investigated the prognostic relevance of hs-cTnT for several systolic and diastolic echocardiographic functional parameters of the left and right ventricles. The univariate GEE regression analyses (Table 6) suggested that the biomarker could predict alterations in the left ventricular systolic tissue velocities and the left ventricular diastolic variables. High-sensitivity cardiac troponin T failed to predict the deterioration in the right ventricular systolic parameters, left ventricular ejection fraction, or the incidence of left ventricular dysfunction (Table 6).

In the multivariate GEE linear regression models—adjusted for age, cancer stage (advanced vs. early), type of systemic therapy (anthracycline, trastuzumab, both or other), HFA-ICOS cardiotoxicity risk level and administration of cardioprotective treatment with RAAS inhibitor and/or beta blocker—the biomarker maintained its prognostic significance for the deviation of the LV systolic velocities (Average and Lateral) and the deceleration time of early diastolic transmitral filling flow.

**Figure 3:**
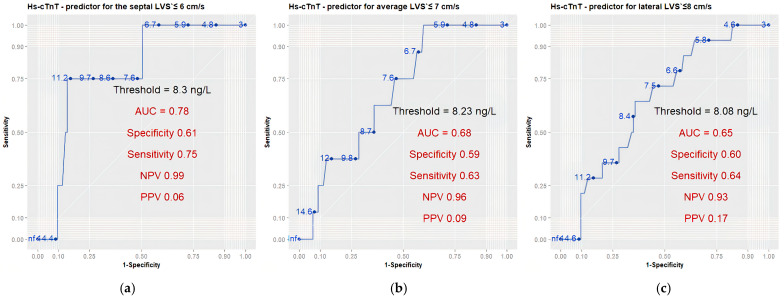
ROC curves for diagnostic ability of hs-cTnT for detecting pathological decline of (**a**) Septal LVS′; (**b**) Average LVS′; (**c**) Lateral LVS′. A pathological decrease is defined as a 15% relative decrease to a value below or equal to 6 cm/s for Septal LVS′ velocity; 7 cm/s for Average LVS′ velocity; 8 cm/s for Lateral LVS′ velocity. Abbreviations in the figure: LVS′—left ventricular systolic tissue velocity; ROC—receiver operating characteristic; AUC—area under the curve; NPV—negative predictive value; PPV—positive predictive value.

The ROC analysis indicated that hs-cTnT was moderately effective in identifying a decrease in Septal LVS′ < 6 cm/s (AUC 0.78; 95% CI: 0.59–0.98), but showed limited capacity to detect a reduction in Average LVS′ < 7 cm/s (AUC 0.68; 95% CI: 0.53–0.84) and Lateral LVS′ < 8 cm/s (AUC 0.65; 95% CI: 0.51–0.78) (Figure 3 and Table 7). The biomarker—at thresholds of 8.3 ng/L, 8.23 ng/L, and 8.08 ng/L—exhibited a high negative predictive value for the pathological decline in the Septal, Average, and Lateral LV systolic velocities, respectively (Figure 3 and Table 7).

## 4. Discussion

The main findings of our study demonstrate that hs-cTnT concentration increases following systemic oncological treatment and might predict the deterioration of some systolic and diastolic parameters of LV function. We further established factors, different from cancer therapy, that could contribute to the biomarker elevation and should be taken into account when interpreting the significance of hs-cTnT for patients’ prognosis.

### 4.1. Early Changes in hs-cTnT Concentration

Our findings indicate that hs-cTnT levels increase early following antineoplastic therapy. Statistically significant increases occurred at the 3rd (β = 1.2; 95% CI: 0.36, 2.0; *p* = 0.005) and 6th (β = 2.3; 95% CI: 0.36, 4.3; *p* = 0.02) post-treatment months (Figure 1). The increase likely reflects the completion of chemotherapy cycles by this time point, with a median treatment duration of 106 (IQR 65, 116) days (Table 2). Another possibility is that the most hs-cTnT samples were obtained within the initial 6 months (Table 4). It is logical to assume that the alteration in hs-cTnT was a consequence of chemotherapy, rather than an effect of HER2-targeted therapy, which was administered later with a median duration of 454 (IQR 365, 514) days (Table 2).

For over 15 years, research has been focused on the efficacy of high-sensitivity cardiac troponins in detecting myocardial injury resulting from various systemic antineoplastic therapies [9,10,11,14,15,16,17,18,19,20,21,22,26,27,28]. In support of our findings, most studies have observed an early increase in high-sensitivity cardiac troponins during or immediately after cancer treatment, with peak levels occurring 3 to 6 months after chemotherapy initiation [10,11,13,15,16,18,21,22]. The vast majority of evidence attributed the elevated hs-cTnT levels to anthracycline-induced myocardial damage [11,14,15,19,22]. Demissei et al. in 2020 found a 4-fold increase in hs-cTnT over six months in patients who received anthracycline, with or without trastuzumab [19]. HER2-targeted therapy has little effect on hsTnT levels, which remained relatively steady throughout the trastuzumab courses, with just a few patients exhibiting high concentrations [11,19].

### 4.2. Rate of Abnormal hs-cTnT Levels

The prevalence of elevated high-sensitivity cardiac troponins associated with cancer treatment varies from 11.4% to 73%, influenced by the specific troponin type (T or I), timing of sample collection, and systemic anti-cancer therapy regimens [10,11,19,27,29]. Upon completion of anthracycline chemotherapy, 25% to 42% of patients exhibit elevated levels of hs-cTnT exceeding 14 ng/L [11,19]. Finke et al., 2021 found a lower rate, 11.4%, of abnormally high hs-cTnT concentration assessed during or after cancer treatment in a large cohort of 930 patients treated with diverse antineoplastic regimens, with only one-fourth of them receiving anthracyclines [27].

Consistent with Finke et al., 2021 [27], we observed a lower incidence rate of 12.5% (*n* = 5) of high hs-cTnT levels exceeding 14 ng/L, likely attributable to the fact that only 48% of our population received anthracycline-containing regimens (Table 2 and Table S1, Figure S1). The hs-cTnT peaked early, within 1 to 3 months, in three patients undergoing anthracycline chemotherapy, and later (at 1 year) in two patients treated with trastuzumab. Despite increased biomarker levels, none of these individuals met our criteria for LV systolic dysfunction.

### 4.3. Factors Influencing hs-cTnT Increase

High-sensitivity cardiac troponins have the potential to detect small amounts of cardiomyocyte damage, induced by multiple conditions that can occur in an oncological environment [5,8]. Pavo et al., 2015 demonstrated elevated hs-cTnT levels before starting antineoplastic therapy, implying cancer-induced cardiomyocyte injury [9]. The researchers correlated the release of serum biomarkers with cancer stage progression and the inflammatory marker C-reactive protein [9]. Malignant cells can initiate a series of processes, encompassing the production of neurohormones and vasoactive peptides, systemic inflammation, heart damage, and cardiac cachexia [9]. Elevated baseline levels of hs-cTns may suggest chemotherapy-vulnerable myocardium, as evidenced by the observation that they can predict future anthracycline-induced decline in LVEF and even cardiotoxicity [16,17,30]. The multifactorial increase in hs-cTns hampers biomarker interpretation in oncological scenarios.

We found that advanced cancer stage could contribute to the hs-cTnT elevation as evidenced by univariate linear regression analysis of all hs-cTnT measurements (β = 3.6; 95% CI: 1.2, 5.9, *p* = 0.003) (Table 5). However, multivariate linear regression analysis demonstrated that advanced cancer stage lacked independent influence on biomarker changes (Figure 2). The regression model indicated that anthracycline treatment (β = 2.8; 95% CI: 0.71, 4.9; *p* = 0.009), a lower glomerular filtration rate (β = −0.14; 95% CI: −0.23, −0.05; *p* = 0.003), and both high (β = 3.7; 95% CI: 0.76, 6.6; *p* = 0.013) and medium (β = 3.6; 95% CI: 1.9, 5.4; *p* < 0.001) levels of cardiotoxicity risk may independently predict an increase in hs-cTnT concentration (Figure 2). The influence of age on the biomarker serum release should not be disregarded, as it affects glomerular filtration rate and cardiotoxicity risk. Decreased renal function may contribute to diminished hs-cTnT clearance, warranting consideration in the analysis of hs-cTnT values [31]. Cardiovascular toxicity risk stratification is a complex variable encompassing various risk factors for future cardiac events, including obesity, smoking, hypertension, and dyslipidaemia [7,32]. Higher baseline hs-cTnT levels may potentially indicate an unrecognised ischaemic heart disease. Cardioprotective treatment with RAAS inhibitors or beta-blockers can prevent troponin rise, especially in patients undergoing anthracycline chemotherapy [6]. Cardio-protection did not influence the change of hs-cTnT in our study, as only half of the patients received a RAAS inhibitor and/or beta-blocker (*n* = 21, 53%). Eight of these patients (39%) were prescribed cardioprotective treatment during the follow-up period.

### 4.4. Predictive Ability of hs-cTnT and Cut-Off Values

Our data indicate that, along with the rise in hs-cTnT, all echocardiographic functional measures showed a statistically significant deviation during the first three to six months (Table 3). The LV and RV systolic tissue velocities, RVFAC, MAPSE, RVMPI (TDI), and LVE/e′ showed the earliest alterations at a median of 3 months. The left ventricular ejection fraction reached its minimum at a later time, with a median of approximately 6 months. The scientific literature confirms LVEF and LV global longitudinal strain (GLS) decline in the first 3 to 6 months after initiating anthracycline chemotherapy, accompanied by an increase in high-sensitivity cardiac troponins [10,15,16,18,20,21,29]. Limited research has assessed variations in additional echocardiographic parameters in connection with changes in high-sensitivity cardiac troponin levels [15,21]. Evidence suggests that LV systolic S′ tissue velocities, PW-derived Tei index, 3DLVGLS, and RVGLS, as well as a rise in hs-cTnT, exhibit early significant deviations following two to four cycles of anthracycline chemotherapy for haematologic malignancies [15,21].

In our analysis of hs-cTnT prognostic performance, we determined that its relative increase can predict decline in LV systolic tissue velocities (both Average LVS′ (β = −0.07, 95% CI: −0.14, 0.00; *p* = 0.036) and Lateral LVS′ (β = −0.09; 95% CI: −0.17, −0.01; *p* = 0.033)) and increases in the diastolic parameter (deceleration time of the LV early diastolic filling flow (β = 1.5; 95% CI: 0.25, 2.7, *p* = 0.018)). The prognostic significance of the biomarker is preserved, even after adjusting for age, cancer stage, type of systemic antineoplastic therapy, and cardiotoxicity risk level and the presence of cardioprotective treatment (Table 6). The predictive ability of the biomarker for Septal LVS′ exhibited borderline statistical significance (β = −0.06; 95% CI: −0.12, 0.01; *p* = 0.088).

Global longitudinal strain and left ventricular systolic tissue velocities reflect longitudinal systolic myocardial function. Global longitudinal strain is a recommended indicator for detecting early cardiac functional impairment [7]. Despite its potential, this approach remains underutilised in cardiology and particularly within cardio-oncology, due to factors such as unavailability, infeasibility, time constraints, and the necessity for training [33,34]. Serial measurements of GLS are essential, as the clinical interpretation of this parameter depends on its relative decline [7]. Systolic tissue Doppler velocities may serve as a substitute for global longitudinal strain in assessing myocardial function when segmental myocardial performance is not a focus, particularly in the context of diffuse myocardial injury resulting from cancer therapy [24]. Systolic tissue Doppler velocities are easily obtainable estimators with excellent re-producibility—characteristics crucial for monitoring cancer patients over time [35,36]. Ichikawa et al., 2024 reported greater accuracy of Septal LVS′ ≤ 6.85 cm/s (AUC 0.73) compared with LVGLS < 17.6% (AUC 0.68) for predicting CTRCD with a similar temporal variation in the parameters [37]. Our study failed to demonstrate the hs-cTnT capacity for prognosticating the LVEF decrease or the more global outcome of left ventricular systolic dysfunction incidence (Table 6).

Scientific data exist on the ability of high-sensitivity cardiac troponins to predict cardiotoxicity, defined by LVEF reduction resulting from anthracycline-induced damage [10,12,13,14,17,18,19,20,27]. For HER2-targeted therapy-induced cardiomyopathy, post-anthracycline hs-cTnT levels are prognostically important [11]. Evidence suggests that the predictive accuracy of high-sensitivity troponins may be enhanced through the integration of specific echocardiographic parameters, such as LVGLS, LV systolic tissue velocities, or the LV Tei index [13,18,21]. No data is available about the prognostic ability of hs-cTnT for the deterioration of left ventricular systolic tissue velocities or diastolic left ventricular parameters in an oncological context.

According to the 2022 ESC guidelines, cardiac troponin levels beyond the upper limit of normal (above the 99th percentile) indicate cancer therapy-induced myocardial damage and define mild asymptomatic cardiac dysfunction [7]. However, the guidelines indicate that reliable cut-off values for predicting and detecting cardiac dysfunction have yet to be established [7]. The documented thresholds in the literature depend on the hsTn type (I or T), antineoplastic regimens, and the timing of the samples in relation to chemotherapy courses.

Two large studies confirmed that levels of hs-cTnT above the upper reference limit of 14 ng/L can predict the development of cardiomyopathy, indicating a 2- to 3.6-fold increased risk in patients undergoing trastuzumab treatment [11,19]. Both studies measured hs-cTnT concentrations after anthracycline chemotherapy; one (the HERA study) defined a threshold of 15.3 ng/L with suboptimal prognostic accuracy (AUC 0.615; 95% CI: 0.515, 0.71), and the other reported a threshold of 14 ng/L (specificity 62.5%, sensitivity 60.3%, PPV 6.5%, NPV 87.5%) with a high negative and low positive predictive value for future LVEF decline [11,19]. A recent meta-analysis, including studies on anthracyclines, trastuzumab, immune checkpoint inhibitors, and radio-therapy, also found that abnormally high biomarker concentrations could detect CTRCD. The performance of hs-cTnT was superior (AUC 0.9; 95% CI 0.87–0.92) when measured earlier, during the initial 3 to 6 months of cancer treatment [12].

Other research revealed lower thresholds of hs-cTnT which could provide clinical information. A cut-off value of 8 ng/L in the third month of anthracycline treatment had a 66.7% sensitivity and 67.9% specificity for predicting CTRCD at 6 months (54.5% PPV, 87.8% NPV) [20]. Levels of hs-cTnT ≥ 7 ng/L were independent predictors of all-cause mortality in a large, heterogeneous study in terms of malignancies examined (breast and gastrointestinal cancers, multiple myeloma, and melanoma) [28]. Compared to the values recorded during the oncological treatment, Bannister et al., 2023 demonstrated that the baseline hs-cTnT level above 10.5 ng/L was the most accurate predictor of anthracycline-induced cardiac dysfunction (AUC 0.75; sensitivity 75%; specificity 80%) [16]. Research has documented the significance of relative increases in the biomarker rather than the absolute cut-off values [13,14,38].

We established hs-cTnT thresholds of 8.3 ng/L, 8.23 ng/L, and 8.08 ng/L to predict a decrease in the Septal, Average, and Lateral LV systolic tissue velocities, with high negative predictive values (0.99, 0.96, and 0.93, respectively) but low positive predictive values (0.06, 0.09, and 0.17) (Figure 3, Table 7). Biomarker levels below 8 ng/L could rule out future deterioration of tissue Doppler systolic velocities and longitudinal left ventricular systolic function. Due to the low positive predictive value, the defined threshold for high-sensitivity troponin would not aid in identifying declines in functional echocardiographic parameters. Our results align closely with those of Bisoc et al., 2020 and Zhang et al., 2017, who established hs-cTnT cut-offs of 8 ng/L and 7.5 ng/L following three months of anthracycline therapy [20,21]. Literature data show that high-sensitivity troponins have a higher negative predictive value than positive predictive value for the occurrence of cardiotoxicity [10,12,13,17,18,19,21,38,39]. This fact corresponds to our findings. In support of this, Demissei et al., 2020 observed that post-anthracycline therapy hs-cTnT levels below 5 ng/L could predict absence from cardiotoxicity at 1 year with absolute accuracy (100%) [19].

### 4.5. Strengths and Limitations of the Study

The principal disadvantage of our study is its small sample size, which is predominantly comprised of female patients, and thus prevents the generalisability of the findings. The population consisted of limited types of cancer and varied in terms of the cancer stage and antineoplastic agents used. The observation periods for individual patients were not uniform. While most patients underwent the hs-cTnT assessment during the initial six months, only half of the cohort had data extending beyond one year. These constraints may have led to the underestimation of the clinical and echocardiographic outcomes. Due to the inability to measure LV and RV GLS, the study failed to assess how these deformational myocardial indices evolved in comparison to systolic tissue Doppler velocities or identify the rate of subclinical myocardial dysfunction as specified in the 2022 cardio-oncology recommendations [7]. The dynamics of the monitored parameters result from the influence of oncological treatment and also reflect the effects of cardioprotective medications administered to nearly half of the population.

Considering all of the study’s limitations, it depicts a realistic clinical scenario in which cancer therapy includes more than one potentially cardiotoxic agent. We observed a sustained increase in hs-cTnT over the first six months following the beginning of chemotherapy, which included various antineoplastic drugs and represented a real clinical scenario. Minor hs-cTnT elevations may indicate myocardial damage correlated with a reduction in LV systolic tissue velocities. This observation may support the use of these conventional and readily accessible echocardiographic parameters for the routine assessment of LV function. Furthermore, we showed that factors other than chemotherapy, such as age, renal function, and cardiotoxicity risk level, could contribute to the hs-cTnT increase. Although we could not prove that advanced cancer stage is an independent factor for hs-cTnT elevation, the possibility of a causal relationship should be considered. The biomarker’s high sensitivity calls for cautious clinical interpretation of its levels and underscores the significance of continuous surveillance and baseline assessment. We identified a threshold value for hs-cTnT slightly above 8 ng/L, below which longitudinal LV function is not expected to deteriorate—finding that necessitates validation in larger patient cohorts. Future studies may focus on developing a model that calculates the future risk of cardiomyopathy by combining echocardiographic variables and hs-cTnT.

## 5. Conclusions

Numerous factors may influence the variations in hs-cTnT levels. According to our results, hs-cTnT levels depend not only on anthracycline chemotherapy but also on renal function and the baseline cardiovascular risk for cardiotoxicity development. These findings align with the 2022 guidelines on cardio-oncology, which recommends for baseline and ongoing monitoring of the biomarker, considering individual patient characteristics and comorbidities. Early relative increase in hs-cTnT, although not reaching abnormal levels, may serve as a predictor of the decline in LV systolic tissue velocities indicative of longitudinal LV function. The established cut-off value of approximately 8 ng/L may assist in the identification of patients who are not at risk for a decline in LV longitudinal systolic function and necessitate additional scientific validation in a larger study population. Future research can explore translating these findings into a prediction model for cardiomyopathy and heart failure development.

## Figures and Tables

**Table 1:** Demographic and clinical characteristics of the study population.

Characteristic	*n* = 40 ^1^
Age, yrs	
Mean, (SD)	54, (12)
Min, Max	31, 74
Gender	
Female	36 (90%)
Male	4 (10%)
Smokers (active or former)	18 (45%)
BMI, mean (SD) kg/m^2^	26.2, (3.9)
Diabetes	4 (10%)
Hypertension	17 (43%)
Dyslipidaemia	5 (13%)
eGFR, mean (SD), min mL/min/1.73 m^2^	97.3 (16.4)
Haemoglobin, mean (SD), min g/L	131 (10.5)
Cardioprotective medications	21 (53%)
RAAS inhibitor (RAASi)	18 (45%)
RAASi—started before the recruitment	13 (32,5%)
Beta blockers (BB)	15 (38%)
BB—started before the recruitment	10 (25%)
Beta-blockers + RAAS inhibitors	12 (30%)
BB + RAASi—started before recruitment	2 (5%)
Statins	5 (12.5%)
HFA-ICOS cardiotoxicity risk level (*n* = 31)	
Low	16 (52%)
Medium	10 (32%)
High	5 (16%)
Follow-up time, median, (IQR) days	360, (162, 478)
Number of follow-up visits, median (IQR)	4, (3.75, 5.0)

^1^ *n* (%); SD—Standard Deviation; BMI—Body Mass Index; eGFR—Estimated Glomerular Filtration Rate (CKD-EPI Creatinine Equation (2021)); RAASi—Renin–Angiotensin–Aldosterone System inhibitors; BB—Beta Blockers IQR—Interquartile Range; HFA-ICOS (Heart Failure Association-International Cardio-Oncology Society) Risk Level, Calculated for the Patients Treated with Anthracyclines, HER2-Targeted Therapy, or Bevacizumab.

**Table 2:** Population characteristics regarding the cancer disease and its treatment.

Characteristic	*n* = 40 ^1^
Cancer type	
Breast cancer	31 (77%)
Colon or rectal cancer	7 (18%)
Pancreatic cancer	1 (2.5%)
Stomach cancer	1 (2.5%)
Cancer stage	
I	6 (15%)
II	16 (40%)
III	11 (27.5%)
IV	7 (17.5%)
Chemotherapy	
Adjuvant	26 (65%)
Neoadjuvant + adjuvant	2 (5.0%)
Neoadjuvant	12 (30%)
Previous chemotherapy	4 (10%)
Previous chest radiotherapy	3 (7.5%)
Drugs in the systemic treatment protocols	
Anthracyclines ^2^	19 (48%)
Cumulative doxorubicin equivalent dose per BSA, mean, (SD) mg/m^2^	123, (43)
Trastuzumab	6 (15%)
Pertuzumab + trastuzumab	4 (10%)
Bevacizumab	4 (10%)
5-Fluorouracil/capecitabine	9 (22.5%)
Taxane	31 (78%)
Cyclophosphamide	21 (53%)
Endocrine therapy	17 (43%)
Types of systemic cancer treatment	
Anthracycline-based chemotherapy	17 (43%)
Anthracyclines + trastuzumab	2 (5.0%)
Non-anthracycline-based chemotherapy + trastuzumab	8 (20%)
Other chemotherapy regimens	13 (33%)
Chemotherapy duration, median (IQR), days	106 (65, 116)
HER2-targeted therapy completion on median (IQR) day	454 (365, 514)
Radiotherapy	16 (40%)
Left-chest radiotherapy	8 (20%)
Total dose > 50 Gy	4 (10%)
Radiotherapy completion on median (IQR) day	183 (155, 270)

^1^ *n* (%); ^2^ Epirubicin; BSA—Body Surface Area; SD—Standard Deviation; IQR—Interquartile Range.

**Table 3:** Baseline functional systolic and diastolic echocardiographic parameters and their maximum deviations.

Echocardiographic Functional Parameters Baseline vs. Maximum Deviations, *n* = 40			*p* Value
LVEF, %	Baseline	Minimal	*p* < 0.001 ^1^
Mean, (SD)	64.6, (4.6)	57, (6)	
Min, Max	57.0, 75.0	44, 67	
Median time (IQR), days	-	171 (85, 233)	
MAPSE, mm	Baseline	Minimal	*p* < 0.001 ^1^
Mean, (SD)	12.94, (1.74)	11.25 (1.648)	
Median time (IQR), days	-	85 (29, 171)	
Septal LVS′, cm/s	Baseline	Minimal	*p* < 0.001 ^2^
Median (IQR)	8.00 (7.00, 8.90)	6.80 (6.00, 7.00)	
Median time (IQR), days		85 (29, 171)	
Lateral LVS′, cm/s	Baseline	Minimal	*p* < 0.001 ^2^
Median (IQR)	9.20 (8.30, 10.50)	7.80 (7.30, 8.50)	
Median time (IQR), days		85 (85, 171)	
Average LVS′, cm/s	Baseline	Minimal	*p* < 0.001 ^2^
Median (IQR)	8.50 (8.00, 9.50)	7.40 (6.85, 8.10)	
Median time (IQR), days		85 (57, 171)	
LVMPI (PW)	Baseline	Maximum	*p* < 0.001 ^2^
Mean, (SD)	0.43, (0.10)	0.54, (0.12)	
Median time (IQR), days		128 (29, 374)	
RVFAC, %	Baseline	Minimal	*p* < 0.001 ^1^
Mean, (SD)	46, (6)	38.6, (5.2)	
Median time (IQR), days		85 (85, 171)	
RVS′, cm/s	Baseline	Minimal	*p* < 0.001 ^1^
Mean, (SD)	12.53, (2.16)	10.60, (1.66)	
Median time (IQR), days		85 (29, 374)	
TAPSE, mm	Baseline	Minimal	*p* < 0.001 ^1^
Mean, (SD)	19.2, (3.3)	16.56 (3.12)	
Median time (IQR), days		128 (85, 171)	
RVMPI (TDI)	Baseline	Maximum	*p* = 0.011 ^2^
Median (IQR)	0.38 (0.32, 0.51)	0.48 (0.37, 0.57)	
Median time (IQR), days		85 (29, 107)	
LVe′, cm/s	Baseline	Minimum	*p* < 0.001 ^2^
Median (IQR)	9.80 (8.80, 11.30)	8.30 (7.20, 10.50)	
Median time (IQR), days		171 (85, 213)	
LV E/e′	Baseline	Maximum	*p* < 0.001 ^2^
Median (IQR)	7.95 (6.40, 8.90)	8.45 (7.40, 9.60)	
Median time (IQR), days		85 (85, 171)	
DecT, msec	Baseline	Maximum	*p* < 0.001 ^2^
Median (IQR)	200 (170, 218)	231 (219, 260)	
Median time (IQR), days		171 (71, 284)	

LVEF—Left Ventricular Ejection Fraction; MAPSE—Mitral Annular Plane Systolic Excursion; LV—Left Ventricular; LVS′—LV Systolic Tissue Velocity; LVMPI (PW)—Left Ventricular Myocardial Performance Index, measured by pulsed wave Doppler; RV—Right Ventricular; TAPSE—Tricuspid Annular Plane Systolic Excursion; RVMPI (TDI)—Right Ventricular Myocardial Performance Index, by tissue Doppler imaging; ^1^ paired *t*-test; ^2^ paired Wilcoxon signed-rank test.

**Table 4:** Median, minimal, and maximal values of hs-cTnT at each visit.

hs-cTnT	Baseline *n* = 40	1st Month *n* = 37	3rd Month *n* = 29	6th Month *n* = 14	9th Month *n* = 6	12th Month *n* = 5
Median (IQR)	6.8 (4.8, 9.6)	6.6 (5.9, 10.3)	8.3 (6.5, 10.0)	8.9 (7.0, 10.9)	6.9 (6.3, 8.4)	9.1 (5.6, 14.6)
Min, Max	3.0, 30.2	3.4, 15.3	3.2, 24.3	5.4, 25.1	5.6, 10.2	3.7, 16.7

hs-cTnT—High-Sensitivity Cardiac Troponin T; *n*—Number of Patients; IQR—Interquartile Range.

**Table 5:** Univariate GEE linear regression analysis for prediction of hs-cTnT change.

Prognostic Factors	Beta	95% CI	*p*-Value
Age	0.19	0.07, 0.31	0.002
Cancer therapy			
Trastuzumab	1.3	−3.4, 0.81	0.2
Anthracyclines	2.2	−0.38, 4.7	0.095
Other	−1.0	−3.2, 1.3	0.4
Cardioprotective treatment	2.1	−0.30, 4.5	0.086
Haemoglobin	−0.05	−0.12, 0.02	0.14
Cancer stage			
Early stage	reference		
Advanced stage	3.6	1.2, 5.9	0.003
eGFR, mL/min/1.73 m^2^	−0.14	0.22, −0.06	<0.001
HFA-ICOS cardiotoxicity risk			
Low	—	—	
Medium	5.2	1.2, 9.1	0.010
High	3.9	2.4, 5.5	<0.001

Abbreviation: CI = Confidence; eGFR—Estimated Glomerular Filtration Rate (CKD-EPI Creatinine Equation (2021)); HFA-ICOS (Heart Failure Association-International Cardio-Oncology Society) risk level—calculated for the patients treated with anthracyclines, HER2-targeted therapy, or bevacizumab.

**Table 6:** Generalised estimating equation linear and logistic regression analyses for prediction of the functional echocardiographic parameters by high-sensitivity cardiac troponin T.

Echocardiographic Variable	Univariate Model			Multivariate Model ^1^		
	hs-cTnT Beta, ng/L	95% CI	*p*	hs-cTnT ^1^ Beta, ng/L	95% CI	*p*
LVEF, %	0.12	−0.09, 0.33	0.3	-	-	-
RVFAC, %	−0.02	−0.30, 0.26	>0.9	-	-	-
Average LVS′, cm/s	−0.07	−0.12, −0.02	0.008	−0.07	−0.14, 0.00	0.036
Septal LVS′, cm/s	−0.06	−0.11, −0.02	0.005	−0.06 ^3^	−0.12, 0.01	0.088
Lateral LVS′, cm/s	−0.08	−0.14, −0.01	0.030	−0.09	−0.17, −0.01	0.033
RVS′, cm/s	−0.08	−0.16, 0.01	0.083	-	-	-
LV e′, cm/s	−0.23	−0.34, −0.12	<0.001	−0.06 ^4^	−0.16, 0.05	0.3
LV E/A	−0.02	−0.03, −0.01	<0.001	0.00	−0.02, 0.03	0.8
LV E/e′	0.24	0.09, 0.39	0.002	0.07 ^5^	−0.02, 0.15	0.11
LV DecT	2.9	1.2, 4.7	<0.001	1.5 ^6^	0.25, 2.7	0.018
LV systolic dysfunction	1.07 ^2^	0.96, 1.19	0.2	-	-	-

Abbreviations: CI = Confidence Interval; ^1^—Adjusted for age, advanced vs. early cancer stage, type of systemic therapy (anthracycline, trastuzumab, both or other), cardiotoxicity risk level and cardioprotective treatment (No or Yes); ^2^—Odds ratio (GEE logistic regression analysis); ^3^—The unique independent predictive factor for Septal LVS′ decrease is the trastuzumab treatment; ^4^—The unique predictive factor is the age (β = −0.13; 95%CI −0.18, −0.08; *p* < 0.001); ^5^— Significant predictive factors are the age (β = 0.13; 95% CI 0.06, 0.20; *p* < 0.001) and the advanced cancer stage (stages III + IV) (β = −2.4; 95% CI −3.7, −1.1; *p* < 0.001); ^6^—Significant predictive factors are: the medium cardiotoxicity risk level (β = −35; 95% CI −55, −15; *p* < 0.001), the advanced cancer stage (β = 33; 95% CI 12, 55; *p* = 0.003) and systemic cancer therapy with trastuzumab (β = −55; 95% CI −81, −29; *p* < 0.001), anthracycline (β = −59; 95%CI −79, −39; *p* < 0.001) or both (β = −93; 95% CI −115, −71; *p* < 0.001).

**Table 7:** ROC analysis for diagnostic ability of hs-cTnT for detecting pathological decline of LV systolic tissue Doppler velocities, defined as a 15% relative decrease to the values noted in the table.

ROC Analysis of hs-cTnT Thresholds for Prediction of Echocardiographic Parameters Abnormal Values						
Echo Parameters	hs-cTnT Threshold ^1^	Specificity ^1^	Sensitivity ^1^	NPV ^1^	PPV ^1^	AUC ^1^
Septal LVS′ ≤ 6 cm/s	8.3 (6.95, 9.67)	0.61 (0.45, 0.73)	0.75 (0.5, 1.0)	0.99 (0.98, 1.0)	0.06 (0.05, 0.08)	0.78 (0.59, 0.98)
Average LVS′ ≤ 7 cm/s	8.23 (6.88, 9.71)	0.59 (0.44, 0.73)	0.63 (0.38, 1.0)	0.96 (0.95, 0.97)	0.09 (0.08, 0.09)	0.68 (0.53, 0.84)
Lateral LVS′ ≤ 8 cm/s	8.08 (6.73, 9.62)	0.60 (0.44, 0.73)	0.64 (0.36, 0.79)	0.93 (0.91, 0.94)	0.17 (0.14, 0.18)	0.65 (0.51, 0.78)

^1^—Value and 95% Confidence Intervals; Abbreviations: NPV—Negative Predictive Value; PPV—Positive Predictive Value; AUC—Area Under the Curve; LVS′—Systolic Tissue Doppler Velocity.

## Data Availability

The data that support the findings of this study are available from the corresponding author upon reasonable request.

## Supplementary Materials

The following supporting information can be downloaded at https://www.mdpi.com/article/10.3390/medicina61111911/s1, Table S1: Chemotherapy regimens, used for initial cancer treatment of the patients in the study; Figure S1. Patient distribution, based on cancer localisation and treatment; Table S2: Baseline left and right heart dimensions; Figure S2. Graphical representation of individual baseline and maximum hs-cTnT values compared to the end of chemotherapy; Figure S3: Maximal deviations of some echocardiographic variables in patients with abnormal elevation of hs-cTnT.

## Abbreviations

The following abbreviations are used in this manuscript:

Average LVS′	average value of Septal and Lateral LVS′
BMI	body mass index
CTRCD	cancer therapy-related cardiac dysfunction
DecT	deceleration time
eGFR	estimated glomerular filtration rate
ESC	European Society of Cardiology
GEE	generalised estimating equations regression analysis
GLS	global longitudinal strain
GLM	generalised linear regression model
HER2	human epidermal growth factor receptor 2
HFA	Heart Failure Association of the European Society of Cardiology
Hs-cTn	high-sensitivity cardiac troponin
IC-OS	International Cardio-Oncology Society
IQR	interquartile range
IVRT	isovolumic relaxation time
Lateral LVS′	tissue Doppler systolic S′ velocity, obtained at the lateral mitral annulus
LAV	left atrial volume
LV	left ventricular
LVe′	early diastolic tissue Doppler e′ velocity
LVEDV	left ventricular end-diastolic volume
LVEF	left ventricular ejection fraction
LVMPI-PW	left ventricular myocardial performance index, measured by PW Doppler
MAPSE	mitral annular plane systolic excursion
RAAS	renin–angiotensin–aldosterone system
RAV	right atrial volume
ROC	receiver operating characteristic analysis
RV	right ventricular
RVA	right ventricular end-diastolic area
RVFAC	right ventricular fractional area changes
RVS′	right ventricular systolic S′ tissue Doppler velocity at the lateral tricuspid annulus
RVMPI-TDI	right ventricular myocardial performance index, measured by tissue Doppler
SD	standard deviation
Septal LVS′	tissue Doppler systolic S′ velocity, obtained at the septal mitral annulus
TAPSE	tricuspid annular plane systolic excursion
VEGF	vascular endothelial growth factor
