# Supplementary material for: High-Sensitivity Troponin T as a Prognostic Factor of Conventional Echocardiographic Parameters in Cancer Patients: A Prospective Observational Study
Source: Medicina (Kaunas). 2025 Oct 24;61(11):1911. doi: 10.3390/medicina61111911 (PMC12654707; doi:10.3390/medicina61111911)
Supplement: Supplementary file 1 [file medicina-61-01911-s001.zip › medicina-3888268-supplementary.pdf]

Supplementary materials

Table S1. Chemotherapy regimens, used for initial cancer treatment of the patients in the study

**Chemotherapy regimens**	***n* = 40**[1]
3EC-3T (3 cycles epirubicin and cyclophosphamide every 3 weeks + 3 cycles docetaxel or paclitaxel every 3weeks)	12 (30%)
4EC-4T (4 cycles epirubicin and cyclophosphamide every 3 weeks + 4 cycles docetaxel or paclitaxel every 3 weeks)	1 (2.5%)
4EC (4 cycles epirubicine and cyclophosphamide every 3 weeks)	1 (2.5%)
3EC-3TH (3 cycles epirubicine and cyclophosphamide every 3 weeks +3 cycles docetaxel or paclitaxel and trastuzumab every 3 weeks	2 (7.5%)
3PTP-3ECP (4 cycles carboplatina, paclitaxel, pembrolizumab + 4 cycles epirubicine, cyclophosphamide, pembrolizumab)	1 (2.5%)
Carboplatina/Paclitaxel – 4 cycles every 3 weeks	1 (2.5%)
4 cycles neoadjuvant chemotherapy Carboplatina/Paclitaxel + adjuvant 3EC	1 (2.5%)
DC (4 cycles docetaxel and cyclophosphamide)	4 (10%)
Docetaxel – 4 cycles docetaxel	1 (2.5%)
ECF (epirubicin, cisplatina, 5-fluorouracil) – 1 cycle	1 (2.5%)
FOLFOX4 (leucovorin calcium, fluorouracil, and oxaliplatin) – 6 cycles every 14 days	5 (13%)
FOLFOX4+Bevacizumab	1 (2.5%)
FOLFOX4+Bevacizumab - 6 cycles every 14 days, FOLFIRI (Irinotecan, Leucovorin, 5-Fluorouracil) – 6 cycles every 14 days	1 (2.5%)
TH (docetaxel + trastuzumab) - 4 cycles every 3 weeks	4 (10%)
THP (docetaxel, trastuzumab, and pertuzumab) – 4 cycles every 3 weeks	4 (10%)

[1]*n* (%)

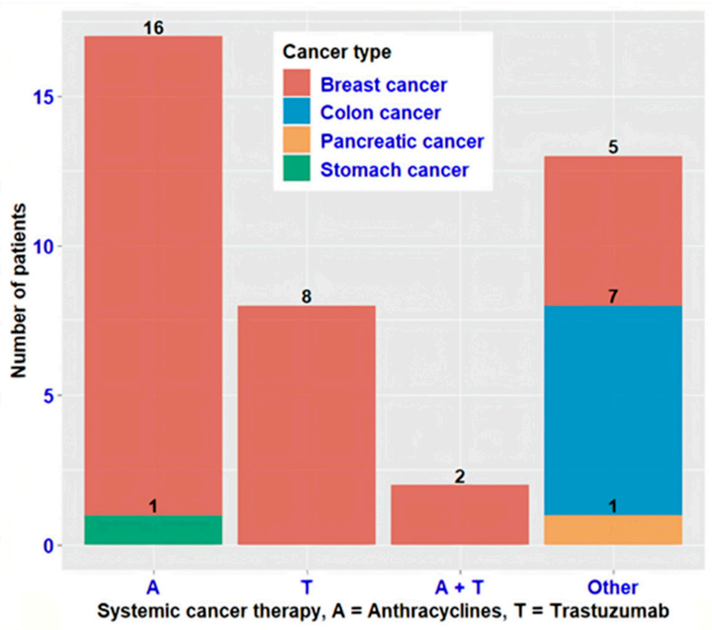

Figure S1. Patient distribution, based on cancer localisation and treatment. Other—systemic antineoplastic treatment, different from anthracycline or trastuzumab, comprising 5-fluorouracil or capecitabine, taxane and/or platina. – to move to supplement

Table S2. Baseline left and right heart dimensions

**Basal values**	***n*= 40**
**LVVi**	
Median (Q1, Q3)	41 (35, 48)
Min, Max	26, 64
**LAVi, ml/m2**	
Median (Q1, Q3)	31 (24, 38)
Min, Max	16, 55
**RVAi, cm2/m2**	
Median (Q1, Q3)	9.31 (7.88, 10.57)
Min, Max	4.65, 13.77
**RAVi**	
Median (Q1, Q3)	22.9 (18.8, 27.7)
Min, Max	12.9, 53.7

LVVi – left ventricular volume indexed for body surface area (BSA); LAVi - left atrial volume indexed for BSA; RVAi – right ventricular area indexed for BSA; RAVi – right atrial volume indexed for BSA;

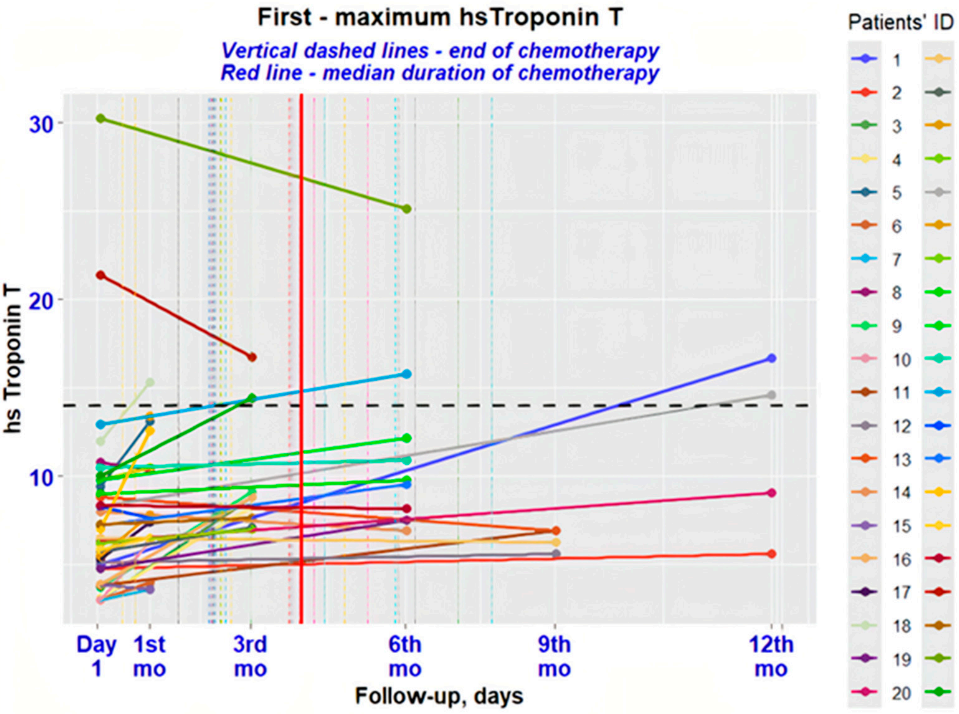

Figure S2. Graphical representation of individual baseline and maximum hs-cTnT values compared to the end of chemotherapy. The black dashed line indicates the upper limit of normal hs-cTnT; the red vertical line shows median chemotherapy duration for the population; and vertical dashed lines indicate individual chemotherapy completion times. – to move to supplement

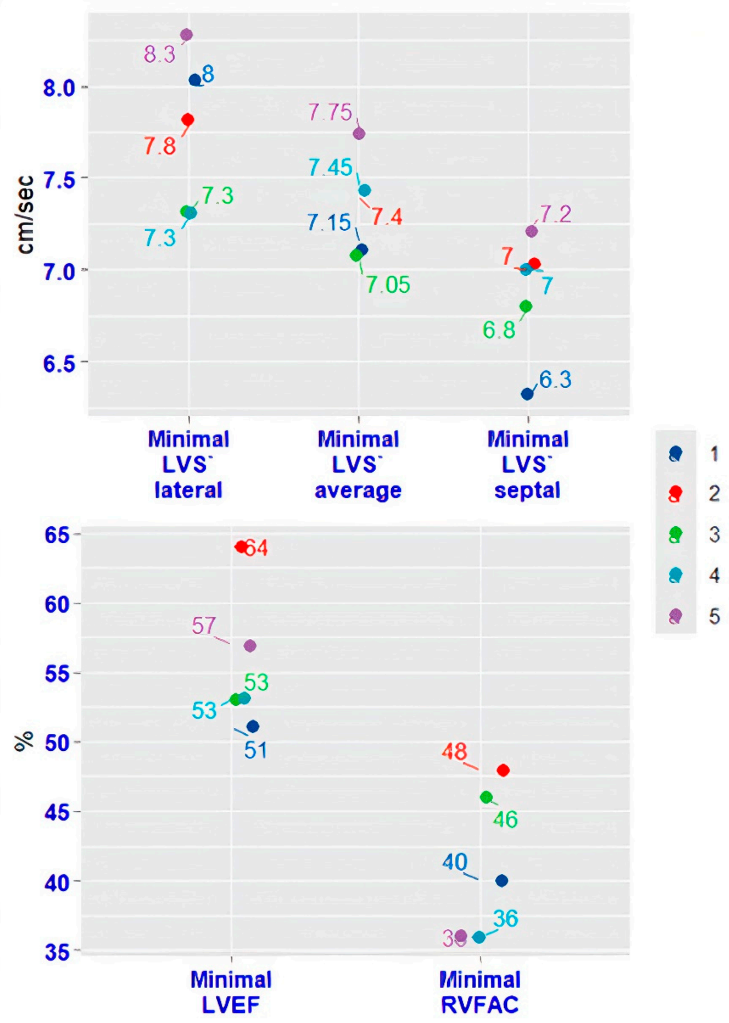

Figure S3. Maximal deviations of some echocardiographic variables in patients with abnormal elevation of hs-cTnT
